# Acute Surgical and Rehabilitation Management of Complex Hand Burns in Combat Casualties

**DOI:** 10.3390/ebj5020011

**Published:** 2024-05-05

**Authors:** Jill M. Cancio, Jonathan B. Lundy, Leopoldo C. Cancio

**Affiliations:** U.S. Army Institute of Surgical Research Burn Center, 3698 Chambers Pass, JBSA, Fort Sam Houston, TX 78234, USA; jlundy1313@gmail.com (J.B.L.); leopoldo.c.cancio.civ@health.mil (L.C.C.)

**Keywords:** hand burns, burn surgery, burn rehabilitation, combat casualty

## Abstract

Burns are inevitable in modern warfare and have comprised between 5% and 20% of battlefield injuries. Involvement of the hands is the leading cause of postburn functional impairment. The purpose of this paper is to provide guidance on aspects of care necessary for the management of complex hand burns in a battlefield setting. Proper assessment and establishment of a comprehensive plan of care at the onset of injury help to ensure optimal functional outcomes in hand function. Basic treatment principles for the acutely burned hand include edema management; early wound coverage, including excision of the burn and skin grafting; early and aggressive hand therapy; and burn-scar contracture mitigation strategies.

## 1. Acute Surgical and Rehabilitation Management of Complex Hand Burns in Combat Casualties

Burns are inevitable in modern warfare [1], comprising between 5% and 20% of battlefield injuries [2,3]. Postburn mortality has declined since World War II, but burns remain a cause of significant long-term functional morbidity [4]. Although nearly 75% of all burns affect less than 20% of the total body surface area (TBSA) [5], they most commonly involve the head and hands [6,7]. Hands are typically exposed and are thus susceptible to thermal injury [1,8,9,10]. In 1982, more than 75% of Israeli military burn causalities from the Lebanese War sustained burns to the hands and face [11]. Eighty percent of burns sustained by combat causalities in Iraq during Operation Iraqi Freedom (OIF) included burns to the hands [8]. Although hand burns from recent conflicts, including Operations Enduring Freedom and Iraqi Freedom (OEF/OIF), account for approximately 5% of all combat casualties [6], the resulting impact on long-term function is substantial [12,13]. The involvement of the hands in burn injury is considered to be the leading cause of functional impairment postburn and is further compounded with larger burns [14,15].

During future multi-domain combat operations with peer and near-peer adversaries, these challenges will be further compounded. Due to loss of air superiority, immediate evacuation from theater, as practiced in the previous OIF/OEF conflicts, may not be possible. As a result, extended care of those who have sustained burn injury, including severe hand burns, will be carried out in a far-forward care environment with limited personnel and resources.

Basic treatment principles for the acutely burned hand include edema management; early wound coverage, including excision of the burn and skin grafting; early and aggressive hand therapy; and burn-scar contracture mitigation strategies [16]. Typically, this care is executed by an intradisciplinary team that specializes in the management of burns. This team includes a burn surgeon, occupational and physical therapists, burn nurses, and optionally a surgeon with hand expertise [16]. The purpose of this paper is to provide guidance on the care of complex hand burns in combat casualties.

## 2. Initial Assessment

Initial assessment of hand burns is challenging even for experienced personnel [17]. Burn depth may evolve over the course of hours to days [18]. Important information to obtain during the initial assessment of the hand includes mechanism of injury, depth of burn, total surface area burned (of the hand and body), as well as any tendon, bone, or joint involvement [19].

**Figure 1:**
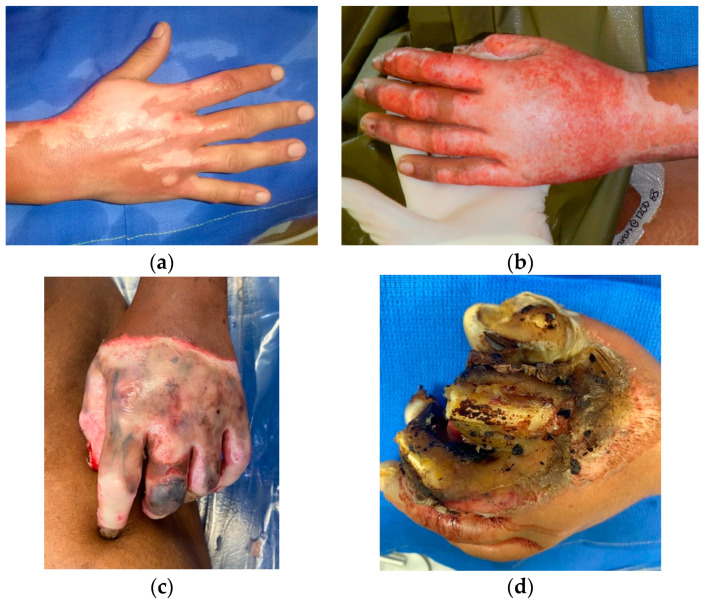
Hand burns at various depths. (**a**) Superficial partial-thickness burn; (**b**) deep partial-thickness burn; (**c**) full-thickness burn; (**d**) “Fourth-degree” burn with extensive destruction of deep structures. Photographs courtesy of the U.S. Army Institute of Surgical Research Burn Center, JBSA Fort Sam Houston, TX, USA.

Early treatment is directed toward resuscitation and prevention of further injury. The wound should be cleansed to remove any chemicals, blisters, and devitalized tissue and to allow for further examination [19]. Physical assessment of the hand includes documentation of the location (i.e., the thicker palmar surface vs. the thinner dorsal surface), staging of the burn (See Figure 1), assessment of sensation and motor innervation, skeletal stability, and circulation. Unfortunately, injury to the hands can be overlooked in the presence of other, life-threatening injuries. Frequent reassessment of the hand is recommended with special attention toward compromises in circulation due to large amounts of edema, circumferential compression by thick eschar, and/or compartment syndrome. Priorities of acute management of hand burns are listed in Table 1.

## 3. Edema Management

**Figure 2:**
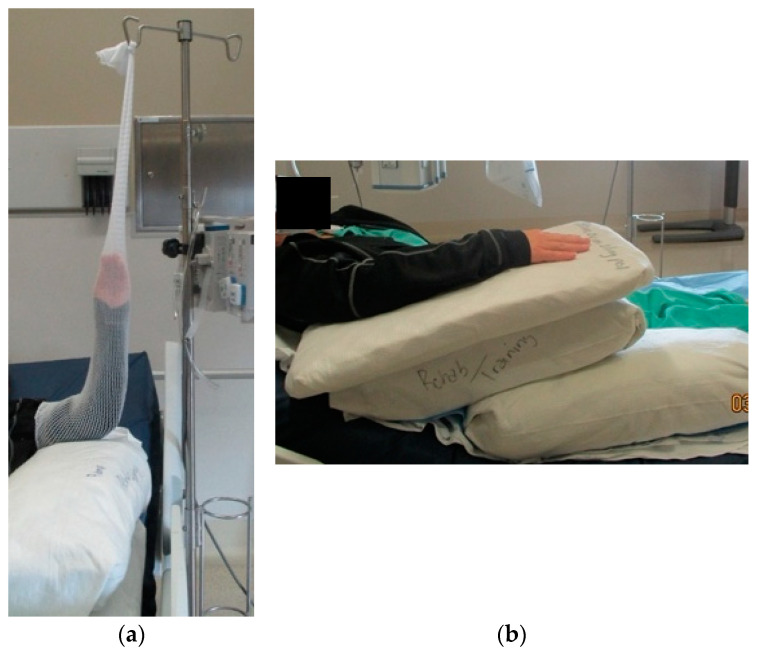
Hand elevation for edema management. Simple elevation techniques for hand-edema management. (**a**) Arm is secured within surgical netting and is attached to IV pole with elbow supported by towel bundle; (**b**) arm is elevated on towel bundles covered in moisture-absorbing pads. Pictures are from the US Army Institute of Surgical Research Burn Center, JBSA Fort Sam Houston, TX, USA.

**Figure 3:**
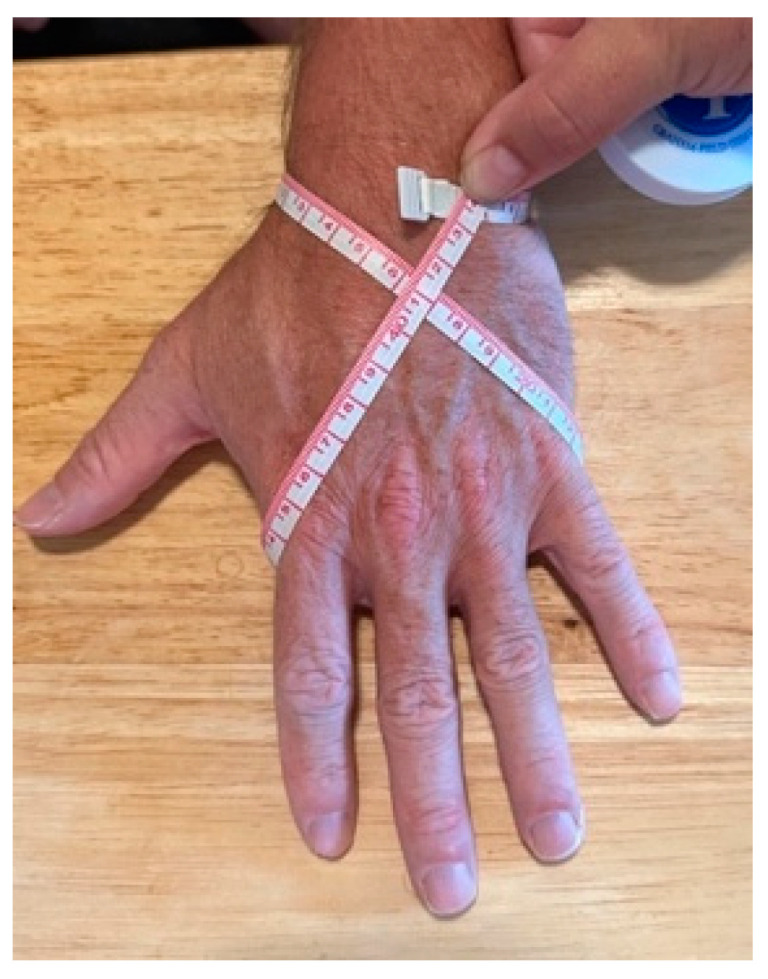
Figure-of-eight hand-edema measurement.

Fluid shifts that occur postburn often cause massive edema, including edema of the hands. Presentation of edema may be delayed, as edema typically peaks 12 to 48 h postburn [20,21]. Elevation of the hands is an effective means of managing edema. Immediate elevation of the extremities, especially burned hands, is essential. The hands should be positioned above the elbows, and the elbows should be positioned at or above the heart. Towel bundles or pillows are readily available items that can be used to elevate the upper extremities (Figure 2a). When more aggressive elevation is required, surgical netting applied to the arm and then attached to an IV pole can be used (Figure 2b). The use of an objective hand-edema measurement supports optimal upper limb positioning. A quick and reliable method is the figure-of-eight technique (Figure 3 and Table 2) [22].

## 4. Eschar and Compartment Syndromes

Eschar and compartment syndromes are limb-threatening conditions which require prompt attention during the resuscitation process. These conditions feature compromise in perfusion to the delicate musculature of the hand and forearm. They may result in catastrophic loss of function or necrosis requiring amputation if not promptly recognized and addressed.

*Extremity eschar syndrome* is the result of deep partial-thickness or full-thickness burns that that encircle a limb or torso, resulting in loss of skin elasticity. As the limb beneath this eschar becomes edematous, there is a gradual loss of adequate perfusion to the underlying tissues.

Monitoring of burned extremities for eschar syndrome should be performed hourly during the first 48 h after injury. It should include Doppler flowmetry of the radial and ulnar arteries, the palmar arch, and digital arteries. Progressive diminution of the Doppler signal is the main diagnostic criterion for this syndrome [23]. Other criteria, used less commonly, include a drop in peripheral saturation of oxygen in affected digits below 90%, or subeschar pressure greater than 30 mmHg. Escharotomy aims to release the tight eschar and restore perfusion. Guidelines available in the literature suggest that the decision to intervene remains a clinical one [24]. In any case, prompt escharotomies are mandatory to preserve the limb and its function [25]. Some authors recommend prophylactic escharotomies in a appropriate clinical setting, such as massive resuscitation needs or severe burns [24].

The procedure can be performed at the bedside with proper sedation, analgesia, and cleansing of the limb. The technique for escharotomies in the upper extremity includes incisions made with a scalpel or electrocautery device on the radial and ulnar side of the limb. The operator should be aware of superficial structures such as (1) the radial nerve on the lateral upper arm; (2) the neurovascular structures within the bicipital groove; (3) the ulnar nerve at the cubital tunnel; and (4) the superficial branch of the radial nerve at the wrist. The incisions should extend beyond the margin of burned tissue onto normal skin and through the full thickness eschar into the subcutaneous tissue. With adequate depth of escharotomy, this will result in a gap between the two edges of the incision as the underlying tissue is allowed to expand. 

**Figure 4:**
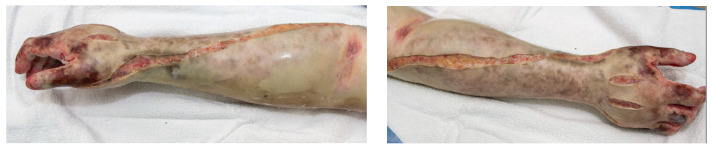
Hand and forearm escharotomy. Pictured is an example of escharotomy incisions on the hand and forearm in an individual who sustained circumferential, full-thickness flame burns. Incisions are placed in the midmedial (ulnar) and midlateral (radial) lines. In this example, pronation of the forearm causes the incisions to appear curved. Photographs courtesy of the U.S. Army Institute of Surgical Research Burn Center, JBSA Fort Sam Houston, TX, USA.

The authors believe that in the setting of circumferential digital burns with a concern for distal perfusion, finger escharotomies should be considered and may be a digit-preserving treatment [26]. Finger escharotomies only require one incision. Care should be taken to place the incision away from the neurovascular bundle. In consideration for protection of sensation for future function, we recommend, if possible, that the incision be placed on the radial aspect of the thumb and ulnar aspect of the index finger to preserve sensation for thumb to index fingertip pinch and grasp. Figure 4 depicts an example of an escharotomy of the hand and fingers.

*Extremity compartment syndrome* is a less common entity that is the result of an increase in pressure within the investing fascia. In a burn center, this is most common after high-voltage electrical injury. After thermal injury, extremity compartment syndrome is usually a result of resuscitation morbidity (indiscriminate, large-volume resuscitation). It may also be seen with an ischemia-reperfusion process (e.g., delayed performance of an escharotomy) or in the context of concomitant mechanical trauma. Upper extremity compartment syndrome can affect the upper arm, forearm, or hand. The diagnosis of compartment syndrome can be made based on clinical suspicion using the classic findings of pain with passive motion, pallor, paresthesia, poikilothermia, and the late and ominous findings of paralysis and pulselessness. Urgent fascial decompression (fasciotomy) is mandatory and should be performed in the operating theater if at all possible.

**Figure 5:**
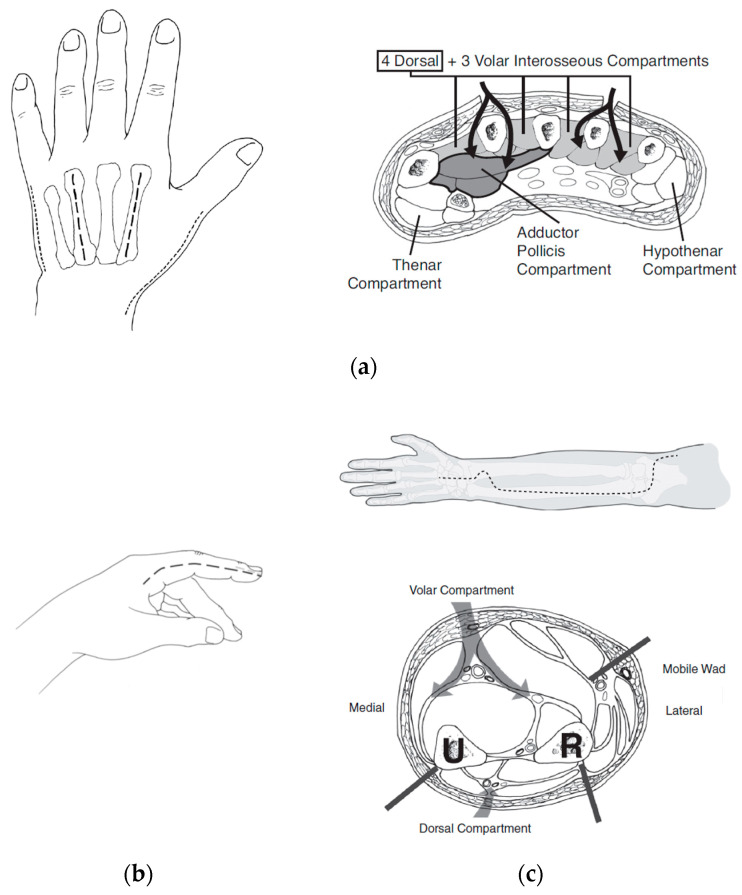
Hand and forearm fasciotomy incision sites. Fasciotomy incision sites for the (**a**) dorsal hand (with associated compartments pictured), (**b**) lateral aspect of digit, and (**c**) forearm (with associated compartments and location of ulnar (U) and radial (R) nerves). Adapted from *Emergency War Surgery, 5th Ed. Borden Institute, 2004* [27].

**Figure 6:**
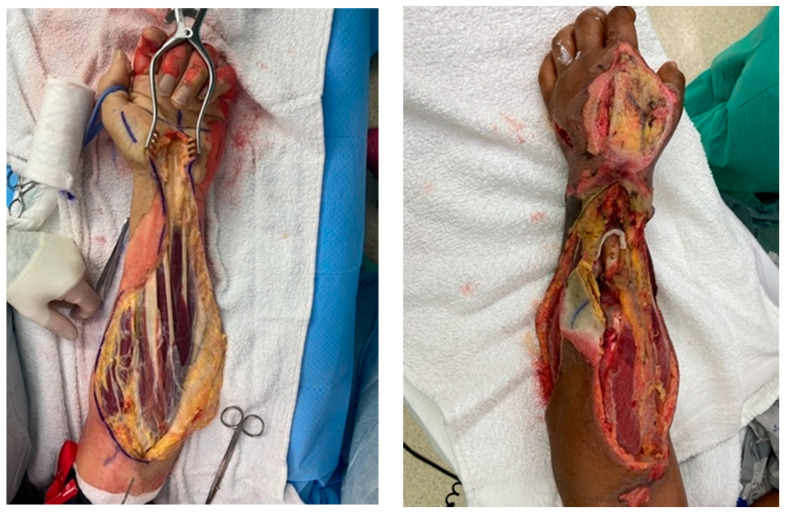
Hand and forearm fasciotomy. Images are of post-fasciotomy procedures completed to hand and forearm in a patient with high-voltage electrical injury. Photographs courtesy of the U.S. Army Institute of Surgical Research Burn Center, JBSA Fort Sam Houston, TX, USA.

Forearm fasciotomies are typically performed using two incisions: a volar S-shaped incision that is carried from just proximal to the elbow to the wrist and onto the volar surface of the hand; and a straight dorsal incision over the extensor muscle bellies. The volar incision involves elevation of skin flaps to adequately decompress the mobile-wad musculature (brachioradialis, extensor carpi radialis longus, and brevis) as well as the superficial and deep volar compartments. Attention should be paid to ensure all muscle fascia is decompressed for each individual muscle belly. This includes the commonly missed pronator quadratus, which rests on the interosseous membrane between the radius and ulna. The elbow and wrist creases should not be crossed with a straight longitudinal incision but should utilize a curvilinear or Z-shape pattern to prevent late contracture development. The incision at the wrist and palmar surface of the hand is used to perform a carpal-tunnel release, ensuring preservation of the palmar cutaneous and recurrent motor branches of the median nerve. Separate incisions are made over the thenar and hypothenar muscle groups of the hand. Incisions on the dorsal hand are made to ensure release of the interosseous muscles. To ensure complete release of all intrinsic muscles of the hand, the muscle fascia at these incisions should be entered and scissors or a clamp used to spread the fascia. Figure 5 depicts the appropriate incisions for hand and forearm fasciotomy incisions. Figure 6 depictures images of fasciotomies after high-voltage electrical injury.

Enzymatic debridement of hand burns is a newer means of restoring and/or maintaining circulation to the hand and digits. Nexobrid^®^ (MediWound Ltd., Yavne, Israel) is a bromelain-based agent used in the enzymatic debridement of eschar in deep partial and full-thickness burns. The enzyme does not penetrate intact keratin; thus, eschar tissue is selectively targeted. This product requires upright refrigerated storage and is packaged in the form of a lyophilized dry powder. The burn wound is cleansed and assessed prior to application of the product and is removed after 4 h. It is important to note that use of this product is painful and often requires the use of opioid medication. The literature suggests that enzymatic debridement of deep burns in the distal upper extremities can prevent eschar syndrome, thus precluding the need for surgical escharotomy [28]. To be sure, use of an enzymatic debridement product requires specialized training focused on proper application and wound care after the product has been removed. Thus, use in a far-forward setting without the capability for follow-on wound care is problematic.

## 5. Wound Care

Following the initial debridement of blisters and loose skin, the burned area should be cleaned with mild soap. Effective wound management includes application of a topical agent with an occlusive or dry protective dressing. To prevent infection and fluid loss and to better manage pain, dressings should be changed daily. Care should be taken to avoid bulky bandaging to the hand. Additionally, each digit should be individually wrapped [1]. This allows for range of motion and functional use of the hands throughout the day and helps to prevent syndactyly of the digits.

Superficial partial-thickness burns will heal without surgical intervention. With consistent wound care and mobilization, these burns are at a very low risk for burn-scar contracture and hypertrophy. Deeper burns that require surgical intervention are dressed in topical agents that provide antimicrobial coverage. Postoperative dressings provide protection of delicate skin grafts and prevent desiccation of the wound. The wound-care plan should be reviewed daily and adjusted depending on the stage of healing. Following closure of burn wounds, topical agents are transitioned to creams to provide moisture and lubrication to the skin. Additional information regarding wound care and commonly used topical agents can be found in the Section 5 of this paper.

## 6. Surgical Management

Traditionally, we have recommended that definitive burn surgery not be performed in the theater of operations because of the complex nature of such care and the need for prolonged, specialized postoperative rehabilitation. Thus, personnel who are assessed as requiring surgery should be evacuated to a burn center outside of the combat zone as soon as possible. However, this is not always possible, particularly in the case of host-nation patients who present for life- and limb-saving care on the battlefield.

If it is determined that the depth of the hand burns is deep partial-thickness or full-thickness in nature, excision and grafting is required to promote timely wound closure. Typically, within 2 to 7 days (depending on medical stability, burn size, and edema levels), the patient is taken to the operating room for tangential excision. Operative management of the hand is usually performed under tourniquet control. Due to the contour of the hand, a Weck or Goulian knife (Teleflex, Inc., Morrisville, NC, USA) is typically used. After the excision is complete, the tourniquet is released, and hemostasis is reached with the use of an electrocautery device. It is important to ensure complete excision and hemostasis are reached to ensure an optimal environment for engrafting of the skin graft.

The type of wound coverage required will depend upon the depth of the excision and availability of donor sites. In smaller burns, excision and grafting can be completed in a single operation. Larger burns or deeper burns may require a staged approach that requires two or more operations. In this case, an allograft or xenograft can be used to protect that excised burn wound until it can be allografted. Typically, dorsal hand burns are grafted with a split-thickness skin graft (STSG). There is no benefit in a thick (0.025 inches) STSG for dorsal hand coverage, and our typical depth of autograft harvest is 0.010 to 0.012 inches. It is thought that sheet grafting provides a superior aesthetic result to a meshed autograft, although there is a risk of seroma or hematoma formation. This can be avoided by pie-crusting the sheet graft or utilizing a 1:1 meshed graft without expanding the interstices [29]. However, use of meshed skin may be unavoidable in cases of larger burns where there are fewer donor sites. An autograft may be secured to the wound bed using absorbable suture, staples, adhesive spray or liquid, adhesive strips, or a combination of these methods based on surgeon experience and material available.

Healing is evaluated in 3 to 5 days, and the plan of care is advanced to achieve functional use. Hand therapy is begun as early as possible but is dependent on the status of the wound. A further description of postoperative management is given in the Section 9 below.

## 7. Complex Coverage Options

Dorsal hand structures can quickly become exposed and are prone to desiccation and further injury due to the relatively thin skin and subcutaneous tissue. When a donor autograft must be used for lifesaving wound coverage over other areas of the body, or there are exposed tendons or other structures, standard STSG use may not be an initial option. In this setting, dermal substitutes/matrices can be used to protect the underlying structures to provide a vascularized dermal replacement and to create a more robust wound bed for later autograft application. There are several dermal substitutes available [30,31,32], including dermal regeneration templates (e.g., Integra bilayer wound matrix; Integra Life Sciences, Plainsboro, NJ, USA) and acellular dermal matrices (e.g., Matriderm; Medskin Solutions Dr. Suwelack AG, Billerbeck, Germany and NovoSorb^®^ Biodegradable Temporising Matrix (BTM) and PolyNovo Biomaterials Pty Ltd., Port Melbourne, Victoria, Australia). Typically, use of these products requires a staged approach: excision and placement of dermal product at the first operation; engraftment of product during a 2–3-week period; and autografting at the second operation. Successful use requires that the wound bed be sterile and free of devitalized tissue. This may not be possible in a battlefield setting. Data from a multi-center, prospective, clinical trial suggest that when managed appropriately, the BTM performs in a relatively robust manner even in the presence of infection [33].

In unusual cases, full-thickness grafts may be used for deep palmar burns [34], especially if the burn is small and with a wound bed favorable for healing. Donor-site locations for these grafts include the proximal wrist crease, hypothenar eminence, medial surface of the proximal arm, and the groin. The medial aspect of the proximal arm is an ideal donor site for a full-thickness graft because the skin is always hairless, and the scar is easily hidden [16].

The combat environment presents many unique challenges in thermal injury management that require ingenuity and creativity to optimize upper-extremity salvage and function. In more severe thermal injury or in soft-tissue disruption with exposure or injury to underlying vascular, musculotendinous, nerve, or boney structures, standard approaches may fall short. A multidisciplinary approach to treatment is most beneficial and where possible the inclusion of the burn surgeon, plastic surgeon, and orthopedic surgeon should occur. Our experience in recent conflicts has shown that this is not always possible. The senior authors have managed host-nation casualties that could not be evacuated to a location with more robust resources. These have included casualties that required complex soft-tissue coverage for limb salvage. Complex coverage options include regional flaps (adjacent tissue), remote pedicled flaps (distant tissue), and free-tissue transfer using microsurgical techniques. Despite the advent of microsurgery in the 1970s, the combat environment is one setting in which pedicled tissue transfer may still be required [35,36].

**Figure 7:**
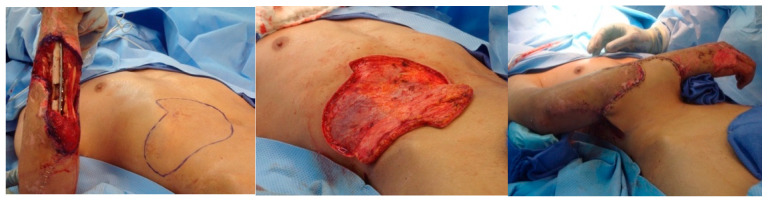
Random abdominal flap for coverage of complex soft tissue injury. Host-nation casualty with open ulna fracture and large soft tissue defect. This casualty was not able to be evacuated out of the combat theater and was managed with serial debridement, open reduction, and internal fixation using a bridge plate and a random abdominal flap for management of the soft tissue deficit. Photographs courtesy of the U.S. Army Institute of Surgical Research Burn Center, JBSA Fort Sam Houston, TX, USA.

**Figure 8:**
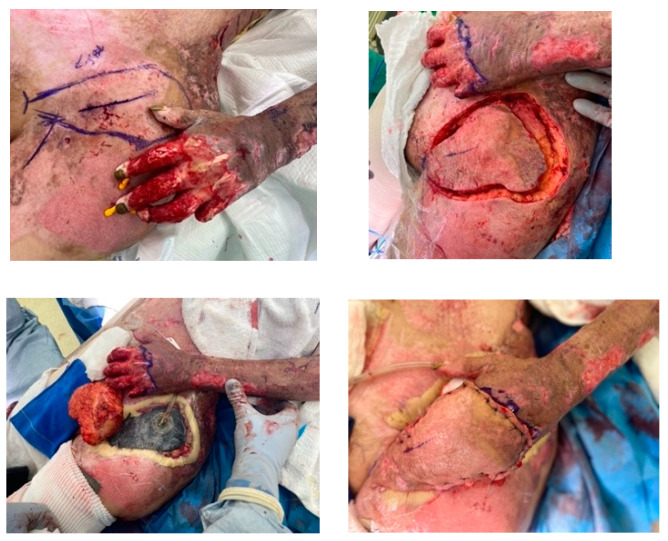
Pedicalized groin flap. Severe thermal injury to the left hand and fingers managed with excision and wound bed preparation followed by groin flap for coverage of remaining extensor mechanism, inter phalangeal joints, and bone. Photographs courtesy of the U.S. Army Institute of Surgical Research Burn Center, JBSA Fort Sam Houston, TX, USA.

**Figure 9:**
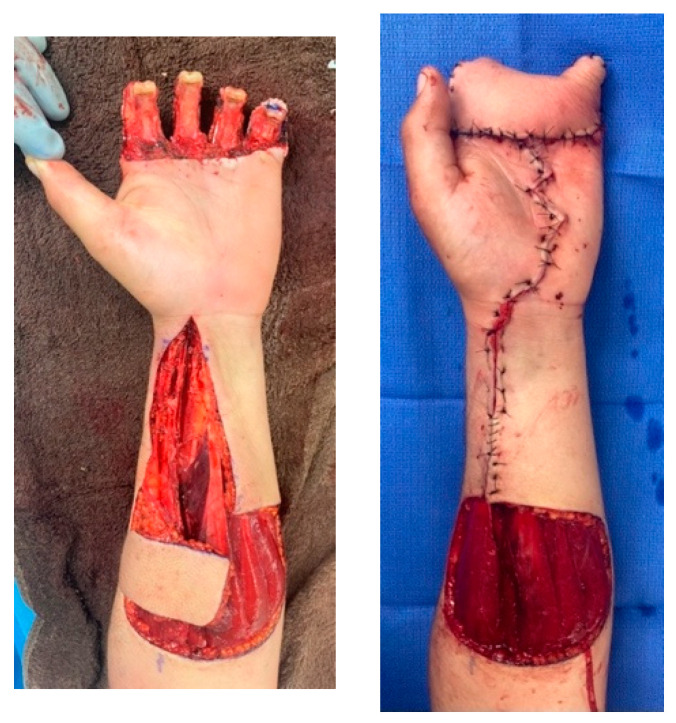
Reverse radial forearm flap. Severe thermal injury to the fingers with loss of middle and distal phalanges and large soft tissue defect over proximal phalanges. This was managed with wound bed preparation and salvage of the residual digits with a reverse radial forearm flap. The syndactylized fingers were later separated and flexor tenodesis performed for restoration of more powerful flexion. Photographs courtesy of the U.S. Army Institute of Surgical Research Burn Center, JBSA Fort Sam Houston, TX, USA.

**Figure 10:**
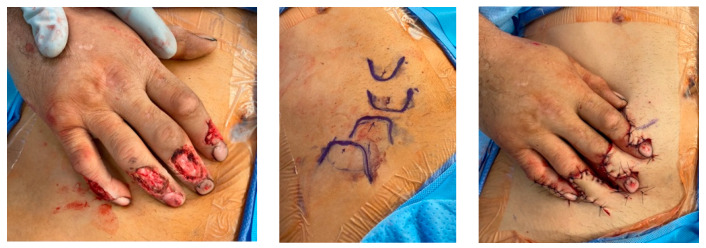
Multiple random abdominal flaps for digits. Soft tissue loss over the dorsal fingers with exposed extensor mechanism due to friction injury after a motorcycle accident treated with serial debridement and the use of multiple random abdominal flaps for coverage. Photographs courtesy of the U.S. Army Institute of Surgical Research Burn Center, JBSA Fort Sam Houston, TX, USA.

The simplest option for coverage of hand and forearm soft-tissue defects or dorsal hand structures requiring complex coverage is the random-pattern abdominal flap. The pedicled abdominal flap is raised in a 1:1 length-to-width ratio and includes skin and subcutaneous fat. This ensures adequate perfusion by the subdermal plexus that supplies this flap, as it is not based on a named, axial vessel. This flap can be taught quite easily and has been performed in our burn center and in the combat setting on multiple occasions. Figure 7 depicts the use of a random abdominal flap for coverage of a complex blast injury to the forearm in a host-nation combat casualty for limb-salvage purposes. Other options for complex coverage include the pedicled groin flap (Figure 8), the reverse radial forearm flap (Figure 9), and multiple random abdominal flaps to address severe finger burns (Figure 10). While the discussion of the principles and techniques for pedicled flap use is beyond the scope of this manuscript, options should be reviewed prior to deployment. Consultation telephonically or using tele/video communication with a hand or plastic surgeon should also be sought where resources allow.

## 8. Kirschner Wires

The purpose of immobilization of the hand after excision and wound coverage is two-fold: to prevent shearing due to inadvertent motion and to counteract the stronger flexion forces that may contribute to a later contracture of the fingers. Several methods have been used to prevent these complications, with the oldest techniques using skeletal traction with banjo, hay-rake, or halo-type splints. As discussed in the rehabilitation portion of this manuscript, modern burn care with splint use is usually sufficient in most hand burns. However, for more severe burns, Kirschner wires (K-wires) may be placed for internal splinting of fingers to prevent inadvertent motion and shearing with resultant loss of skin grafts. There are theoretical benefits to this practice, especially in a markedly disadvantaged digit with little subcutaneous tissue overlying the extensor mechanism. This practice should be guided by a protocol to ensure (1) that wires are not retained for a prolonged period; (2) safety of staff members caring for patients with potentially exposed wires; and (3) prevention of complications such as prolonged immobilization, crossing joints that do not require immobilization, and septic complications such as osteomyelitis or septic arthritis. We typically utilize K-wires for internal splinting in the setting of severe hand burns that have failed initial attempts at coverage when there are exposed structures such as tendons, bones, or joints [37]. This strategy is mainly for the purpose of minimizing the late proximal interphalangeal (PIP) joint contracture with Boutonniere deformity that results from loss of dorsal skin over the joint, shortening of the volar plate, central slip exposure and rupture, and worsening of the PIP flexed posture.

**Figure 11:**
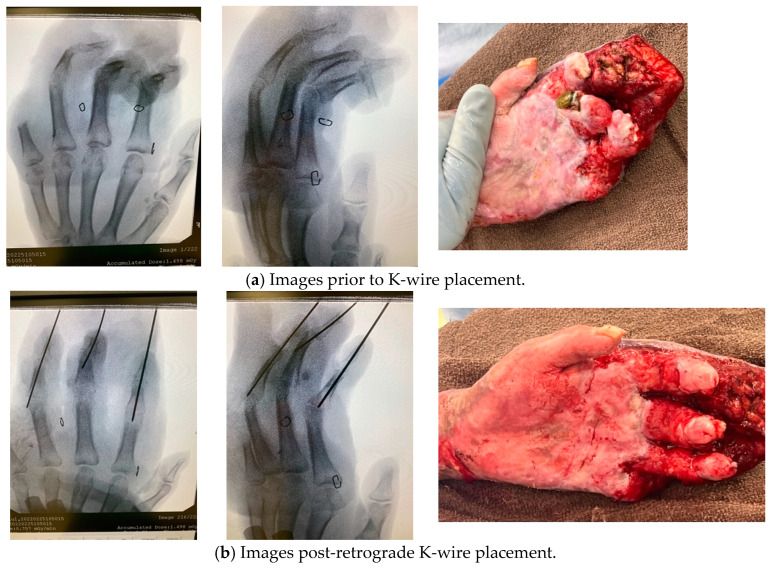
K-wire placement in severely burned digits. Photographs courtesy of the U.S. Army Institute of Surgical Research Burn Center, JBSA Fort Sam Houston, TX, USA.

Wires should be nonthreaded for ease of removal. They can be inserted retrograde from the tip of the finger across the joint or area of concern (most commonly the PIP joint). Antegrade insertion through the MCP joint or soft tissue about the MCP joint should rarely be necessary but occasionally may be in more severe dorsal hand burns. Figure 11 depicts before and after K-wire placement to address severe acute digit contractures in a burned hand with destruction of central slips and exposure of DIP joints. Although ideally wires should be placed using fluoroscopic guidance to ensure optimal placement location, this is not mandatory [38,39]. But imaging should be performed as soon as possible to ensure no complication of wire insertion and to ensure that only the area that requires immobilization is involved. Follow-up imaging should also be performed after removal to rule out complications such as osteomyelitis. Timing of wire removal should be guided based on the adherence and perfusion of the autograft and concern for underlying structures. We recommend that wires should remain in place for a maximum of 3 weeks and an alternative method chosen if longer periods of immobilization are needed thereafter. Some advocate for longer periods of K-wire immobilization, especially in the pediatric population [40]. All members of the treatment team should be made aware of the presence of K-wires and of the plan for removal [41].

## 9. Rehabilitation and Postoperative Management

In a burn center, the involvement of occupational therapy for the management of hand burns is critical. The overall goals of hand therapy include ongoing edema management, maintaining maximal range of motion, protection of skin grafts, fabrication of specialized splints to maintain maximum tissue length, neurological assessment of motor function and sensation, and overall guidance of the recovery of hand function [19]. This section will address rehabilitative factors in management of acute hand burns as well as offer techniques that can be used in an environment with limited resources such as the battlefield.

**Figure 12:**
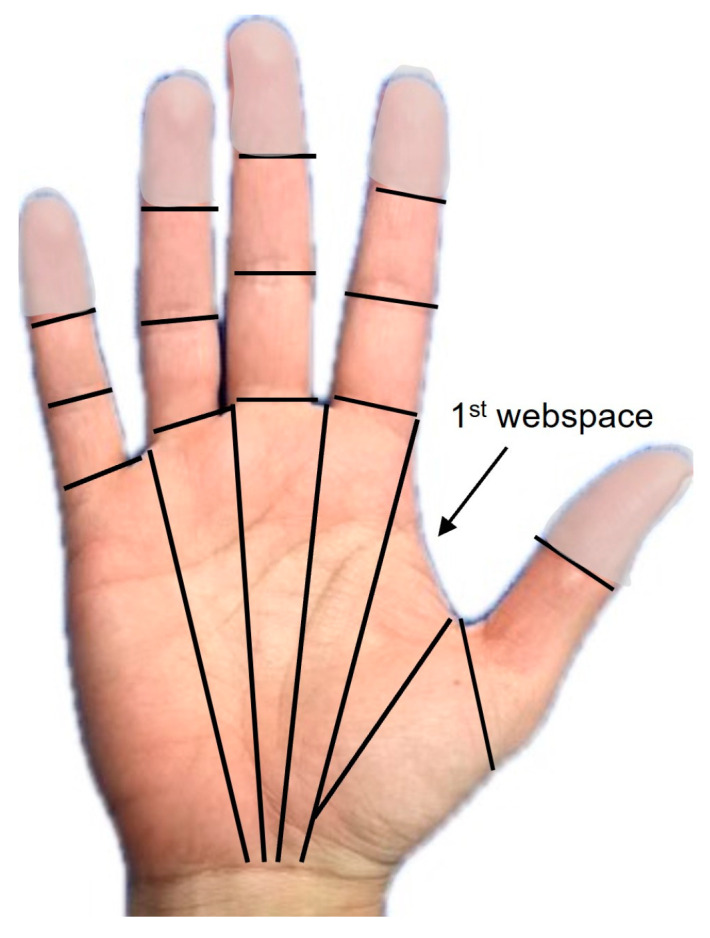
Cutaneous functional units. Location of cutaneous functional units on the palmar surface of the hand.

Tracking the number of involved cutaneous functional units (CFUs) is a useful tool in determining the severity of a hand burn. CFUs are defined as fields of skin associated with motion at adjacent joints [42]. The CFU considers specific skin areas affected by the burn rather than the total body surface area (TBSA) burned. For example, burn injury to the hands only comprises approximately 2–4% of the overall TBSA, but there are over 30 CFUs per hand (Figure 12).

## 10. Positioning of the Hand and Upper Extremity

A common phrase in burn rehabilitation is that “the positioning of comfort will become the position of deformity”. Positioning of the patient after burn injury is fundamental to bringing about the best functional outcomes. This is particularly the case with hand and upper-extremity burns [43,44]. Although challenging to implement in practice, commitment to consistent and effective positioning of the hand throughout all the phases of rehabilitation is helpful to minimizing physical impairment. Positioning and edema management begins upon admission to the burn center.

As mentioned previously, elevation of the hand and upper extremity should occur upon admission to address edema. Elevation of the hand persists during the hospital stay to address changes in edema after surgical intervention and progression through stages of healing. In addition to elevation as an edema-management technique, range of motion and compression are also useful in managing edema. Gentle active range-of-motion (AROM) and tendon-gliding exercises applied during the acute phase of rehabilitation create a muscle-pumping action that is effective in reducing edema and in decreasing tendon adhesions.

## 11. Compression

Compression by means of edema gloves or self-adhesive wrap (Coban) applied to the fingers are common means of managing edema. Edwick et al. [45], found that Coban is superior to edema gloves. Coban should be applied in a distal-to-proximal direction on the digits. We recommend that a strip of Coban be applied between the digit web spaces to aid in edema reduction and prevent syndactyly. During the acute phase, prolonged use of Coban is not recommended with topical agents that require regular wet downs, as this may cause maceration of the skin, or in cases where thicker bandages are necessary. Coban is more useful in later phases in the case of deeper burns after the placement of the STSG. Proper application to allow perfusion of the digits and monitoring for signs of breakdown and/or maceration are necessary.

Identification and use of an objective edema-measurement technique can help support treatment strategies. The figure-of-eight measurement technique (see above) is a valid and reliable method to assess hand and upper-extremity edema [22]. Hand edema should be measured early and consistently in the acute management of hand burns.

## 12. Splinting

**Figure 13:**
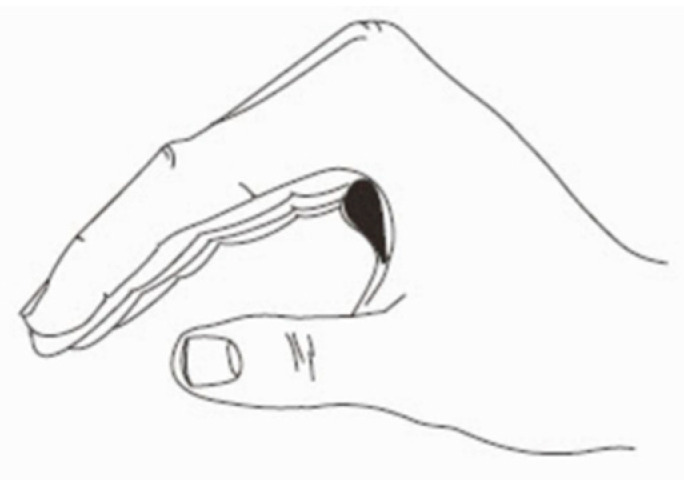
Intrinsic plus or “safe” position. Adapted from *Emergency War Surgery, 5th Ed. Borden Institute, 2004* [27].

**Figure 14:**
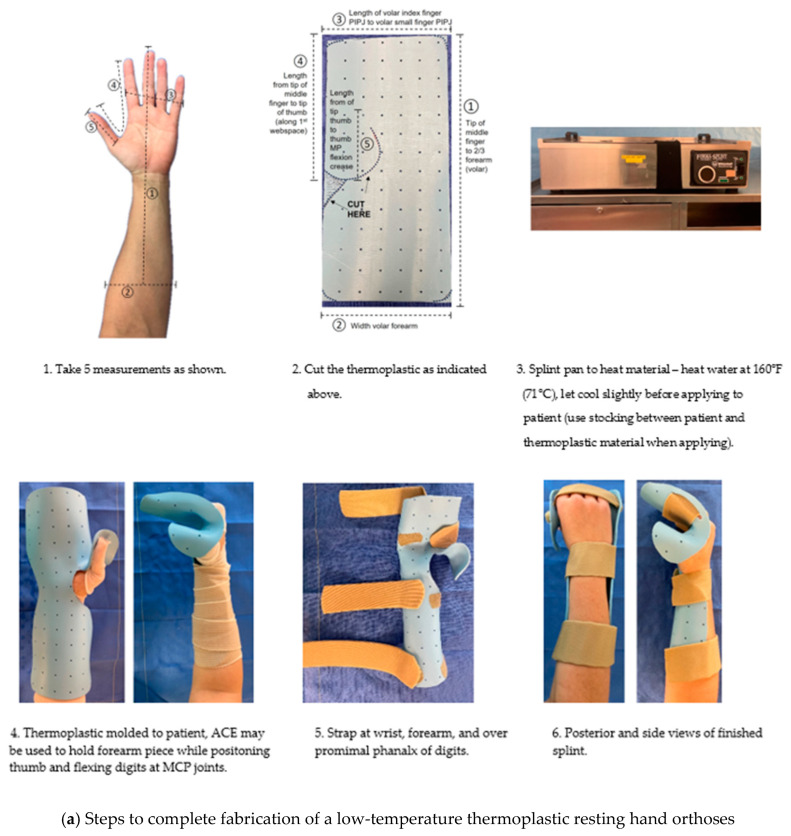

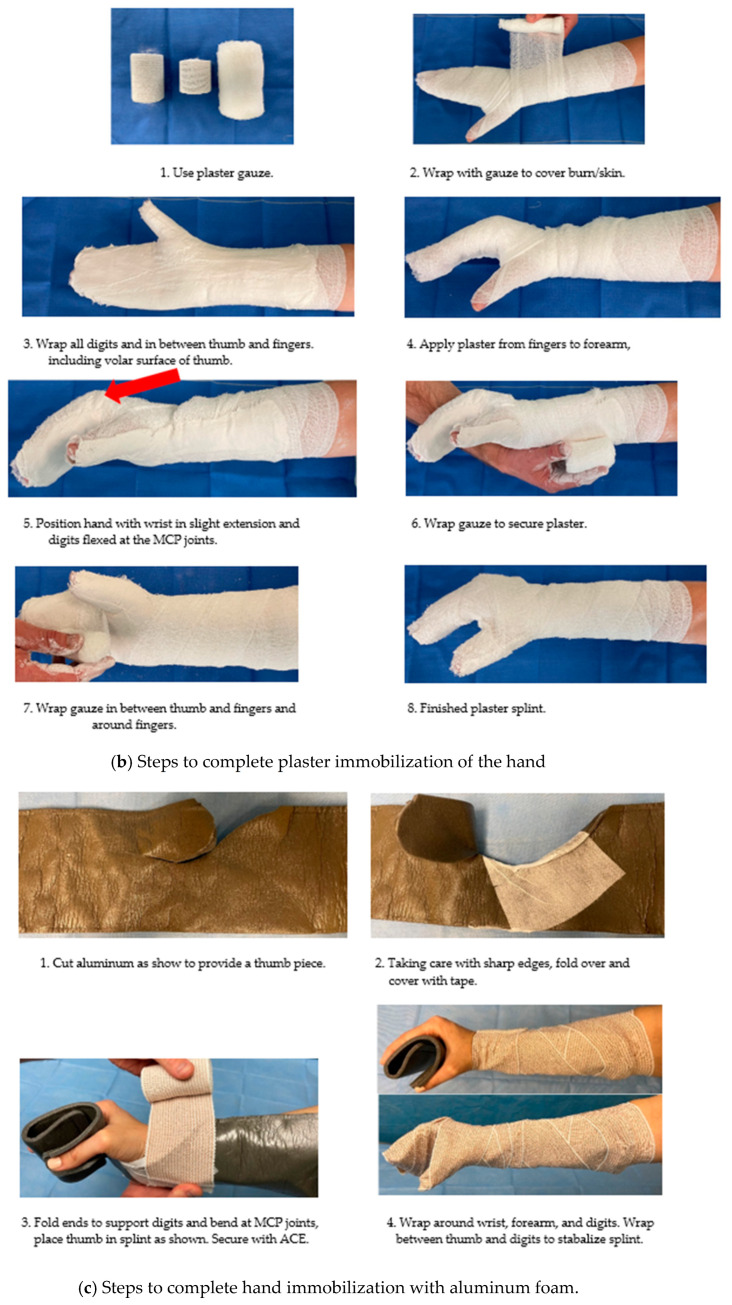
Splint immobilization with Various Materials. Photographs courtesy of the U.S. Army Institute of Surgical Research Burn Center, JBSA Fort Sam Houston, TX, USA.

Positioning the joints of the hand and wrist opposite the direction of skin tightness with the use of orthotic devices is often necessary for the prevention and treatment of burn-scar contracture. Splinting of the hand commonly takes place after STSG procedures for protection of newly placed grafts. Typically, the digits are placed in a “safe” position during the acute phases of care to protect capsular and collateral ligament structures [46]. The safe position is depicted in Figure 13. The wrist is positioned in 20° extension, metacarpophalangeal (MCP) joints of the index to small fingers in 70–90° extension, proximal interphalangeal (PIP) joints and distal interphalangeal (DIP) joints in full extension, and the thumb in palmar abduction. This position helps to prolong the development of stiffness and contracture to the hand. In a burn center, low-temperature thermoplastic and Velcro strapping are used fabricate custom orthoses. In the acute phase, prefabricated burn splints made from thermoplastic are more clinically expedient than custom splints and can generally meet positional needs. In a more austere setting where thermoplastic material may be limited or not available, we recommend the use of plaster or aluminum foam material. In addition to the fabrication of thermoplastic splints, Figure 14 depicts use of these materials to position the hand. These materials may be utilized both postoperatively and in later stages of recovery for nighttime positioning to provide prolonged lengthening of burn scars in a position opposite to the skin tightness. Other custom splints or orthoses that are commonly utilized with burned hands beyond acute management include c-bar orthoses for first webspace contractures and forearm-based static progressive orthoses to address MCP contractures. Further resources for these splinting methods of hand burns are available [47,48].

## 13. Range of Motion and Functional Movement

Hands have the highest incidence of contracture [49]. As such, digit range of motion (ROM) should be employed to prevent and correct burn-scar-related deficits. Prior to initiating a mobilization plan, assessment of function via goniometric measurements should be documented to track decreases and increases in ROM over time. AROM, passive range of motion (PROM), and total active motion (TAM) of digits should be collected based on wound location and burn depth. Documentation of accurate and appropriate finger ROM after a burn injury is important in determining an appropriate treatment program and documenting the burn survivor’s functional status. Richard et al. [50] recommend use of specific goniometric techniques to provide an accurate picture of hand function.

As mentioned previously, in the acute phase of burn rehabilitation, AROM of the hand provides gentle gliding of the tendons to help prevent decreases in ROM due to adhesions as well as treat hand edema. Burn survivors should be encouraged to use their burned hands in the completion of functional tasks after initial injury and to continue throughout all phases of rehabilitation unless contraindicated (i.e., exposed extensor tendon over PIP joint or immediately after the STSG). It is recommended that therapists closely monitor ROM performance and ensure that the scar is blanching during movement to ensure tissue is being lengthened to the greatest extent possible. The term “aggressive ROM” in burn rehabilitation should be interpreted as the use of “low-load, prolonged stress” to the burned skin [51]. The use of prolonged, gentle stress to burned tissue in order to promote tissue lengthening is commonly employed in the burn rehabilitation setting. In the acute phase of burn injury, skin tightness may not be as evident. However, skin tightness and the burn scar must be addressed while they are developing during the proliferative stage of healing, beginning approximately 5–7 days after injury. If the burn survivor is alert and following commands, education on exercise and functional use of hands throughout the day is imperative. It is certain that one rehabilitation session a day with no additional movement is not adequate to prevent contracture. It is imperative the burn survivor be actively engaged in their recovery process.

Sedation during the acute phase of burn care can lead to extended periods of immobility. This places the skin and joints at higher risk of developing contracture. Joint contracture can occur due to capsular and collateral ligament shortening, particularly at the MCP joints. In this case, PROM several times per day is useful. There are no data to inform the precise amount of ROM needed to prevent skin tightness due to burns. Family education regarding completion of ROM several times throughout the day outside of rehabilitation treatments can help to further minimize complications. It is important to consider depth of burn and risk of further damage to joints with deeper burns in the decision to allow others outside of the rehabilitation staff to complete PROM.

## 14. Potential Complications

**Figure 15:**
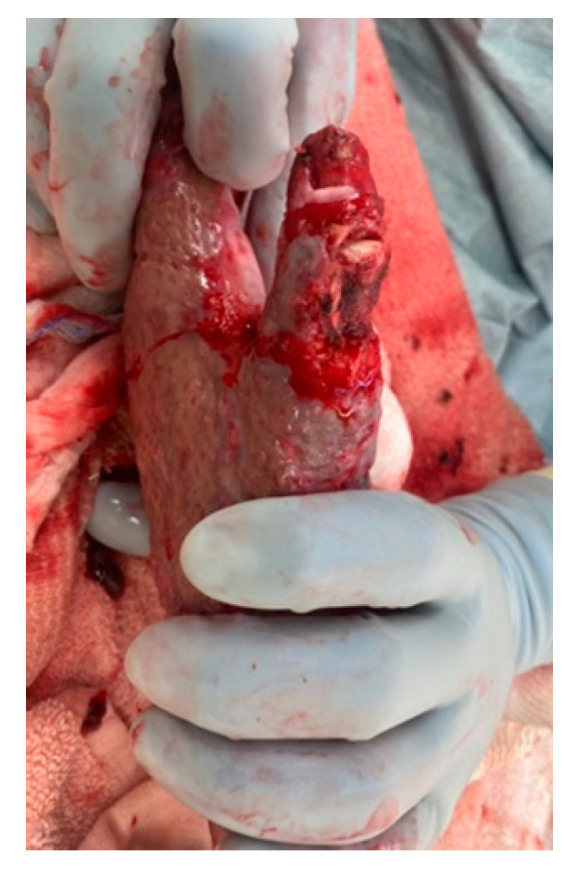
Terminal extensor tendon rupture and exposure of IP joint of thumb. Photograph courtesy of the U.S. Army Institute of Surgical Research Burn Center, JBSA Fort Sam Houston, TX, USA.

Despite our best efforts, deep hand burns place the patient at risk of complications. Table 3 lists common deformities caused by acute hand burns. Exposed tendons are at great risk for drying and rupture. Extensor tendon injury can occur in several locations on the digit. The terminal extensor tendon can be damaged, which can lead to a mallet-finger injury characterized by the loss of extension at the DIP joint. Figure 15 depicts the loss of the extensor mechanism at the terminal insertion and exposed IP joint of the thumb. The central tendon over the PIP joints is the most common site of extensor tendon damage. A boutonnière deformity can result from this type of injury. If these structures are exposed and allowed to dry out, they can easily rupture with forced flexion, specifically composite flexion of the MCP, PIP, and DIPJ together. Visual assessment of the status of the skin is paramount to prevent further injury of these delicate structures. Exposed tendons can be covered with petroleum-jelly-impregnated gauze and the fingers immobilized individually or together.

The accessory collateral ligaments of the MCP and IP joints are also structures at risk of damage. Edema in the digits and hand can force the hand into a claw position with the MCP joints in hyperextension and the PIP and DIP joints in flexion. This position places the collateral ligaments in a relaxed position, putting these structures at risk for contracture if the hand is left in this position for long periods of time. Use of a resting hand splint to place the hand in a “safe” position can help to prevent this contracture.

Thumb function comprises approximately 40% of total hand function. The first webspace is a uniquely endowed anatomic feature that allows for circumduction and opposition and enables activities of pinch and grasp. Burn injury to the thumb represents a much more significant loss than of any other digit. Attention to the positioning of the thumb to allow maximum tissue length of the first webspace (palmar verses radial abduction) after the STSG is critical in preservation of function.

Motor loss as a result of peripheral neuropathy is another complication that can limit active motion of the hand after burn injury [52]. Due to the subtle and progressive onset of neuropathy, it is often overlooked. In the acute phase, neurologic symptoms can be masked by shock, multiple organ failure, or need for ventilatory support and sedation. Neuropathies of the median, ulnar, and/or radial nerves of the hand can cause muscle imbalance, and the lack of movement in the affected area can compound the contracture risk. Early recognition is imperative for successful management. A thorough motor exam by the occupational therapist should take place as soon as possible, with results communicated to the treatment team. Goals for management include prevention of soft-tissue tightness from the muscle imbalance as well as substitution for the motor loss with functional splinting [53].

## 15. Conclusions

Acute treatment of hand burns requires an experienced interdisciplinary team including burn and hand surgeons, occupational therapists, and specialized nurses. Determination of burn depth and monitoring of adequate circulation requires ongoing monitoring throughout the resuscitation phase. Superficial and superficial partial-thickness burns can be treated conservatively with proper wound care and management of ROM through a structured program of functional use. Deep partial and full-thickness burns require early operative management with excision and grafting. Regardless of surgical technique, postoperative management including rehabilitation and wound care is critical in preserving long-term function. Proper assessment and establishment of a comprehensive plan of care at the onset of burn injury helps to ensure optimal functional outcomes in hand function.

## Figures and Tables

**Table 1:** Acute management of hand burns.

1.	Cleanse and debride wounds to evaluate/stage burn.
2.	Elevate burned extremities; assess edema consistently and frequently.
3.	Decompress eschar and compartment syndromes as needed.
4.	Initiate rehabilitation upon admission; focus on positioning, function, and mobility.
5.	Schedule early excision and grafting.
6.	Provide occupational therapy, including early and aggressive range of motion and functional activities.

**Table 2:** Hand figure-of-eight measurement procedure.

Place wrist/digits in neutral and forearm in pronation
2. Start distal to ulnar styloid
3. Move across volar wrist to point distal of radial styloid
4. Run diagonally across dorsal hand
5. Align distal edge of tape over MCP joint flexion crease below small finger
6. Move across palmar aspect of hand with distal tape touching 5th to 2nd finger flexion creases
7. Wrap around 2nd metacarpal head and place diagonally across dorsal hand to starting point
8. Endpoint is intersection of distal edges of the starting and end points of the tape

Adapted from Dewey, W. S.; Hedman, T. L.; Chapman, T. T. et al. *J Burn Care Res.* 2007, *28* (1), 157–162 [22].

**Table 3:** Common deformities after acute hand burns and related structures.

Deformity	Structures Involved	Corrective Treatment
Claw Hand	Contracture of accessory collateral ligaments of MCP (extension) and IP (flexion) joints	Splint in “safe” position with resting hand splint (day and/or nighttime wear schedule)
Boutonnière Deformity	Rupture of central extensor tendon over	Splint affected digit to position PIP joint in full extension (gutter splint)
Mallet-Finger Deformity	Rupture of the extensor tendon at the terminal insertion	Splint affected digit DIP joint in full extension to slight hyperextension (stack splint)
First-Webspace Contracture	Skin of first dorsal and/or palmar webspace	Low-load, prolonged stress of skin opposite to skin tightness (radial or palmar abduction); inclusion of thumb in splint (c-bar)

MCP = metacarpal–phalangeal; IP = interphalangeal; DIP = distal interphalangeal.

## Data Availability

No new data were created or analyzed in this study. Data sharing is not applicable to this article.
